# Announcement of conversion of *Journal of Synchrotron Radiation* to 100% open access from January 2022

**DOI:** 10.1107/S1600577520013922

**Published:** 2020-11-01

**Authors:** Editorial Office

**Affiliations:** a International Union of Crystallography, 5 Abbey Square, Chester, CH1 2HU, United Kingdom

**Keywords:** Journal of Synchrotron Radiation

## Abstract

The proposed conversion of *Journal of Synchrotron Radiation* to 100% open access from January 2022 is announced.

The International Union of Crystallography (IUCr) has concluded that the time for converting *Journal of Synchrotron Radiation* (*JSR*) to become fully open access has arrived. With this issue we are pleased to announce that *JSR* will become a fully open-access journal from the beginning of 2022. More specifically, and with the approval of the IUCr Executive Committee, the decision has been made to convert *JSR* to fully open access from the January 2022 issue.

Full open access will require the contact author of each *accepted* paper (or their institution) to pay an article processing charge (APC). The APC will apply to all *accepted* papers (after reviews are completed), unless a waiver or discount has been agreed between the contact author and the Editorial Office. Authors from developing countries will be able to apply for a full or 50% waiver of the APC. All papers in new *JSR* issues from January 2022 will be made open access and freely available to all researchers (*i.e.* they will be gold open access). The costs of peer review, of journal production, and of online hosting and archiving will be met from the APC.

Further details will be made available as we get nearer to the conversion date, and the following transitional timetable will apply:

(1) Submission for publication from now and through all of 2021 – this will be the open-access transition period, in which publication of conventional behind-pay-wall articles is still possible but open access will be increasingly encouraged. Discounts in the open-access APC will be available where appropriate. Each 2021 *JSR* issue will reprise the announcement of full open access coming to *JSR* for papers published from January 2022.

(2) For papers submitted from 1 October 2021, the contact author will be asked to agree to payment of the open-access APC *if* their article is accepted, unless a waiver or discount has been agreed between the contact author and the Editorial Office.

(3) *JSR* becomes fully open access from the January 2022 issue. Conventional behind-pay-wall articles will no longer be possible, although APC discounts (and sometimes waivers) will still be available.

While this is a major change for *JSR*, presenting both challenges and opportunities, ultimately it is made in the spirit of expansion rather than contraction.

In this connection, we much appreciate the help of *JSR’s* supporting institutions, and we hope other major facilities will also consider supporting *JSR* as the journal of choice for papers focused on synchrotron or FEL-based X-ray structural science by taking up an open-access facility arrangement.

